# Complete Genome Sequence of *Apricot Pseudo-Chlorotic Leaf Spot Virus* from *Prunus persica* in South Korea

**DOI:** 10.1128/MRA.00153-19

**Published:** 2019-05-16

**Authors:** San Yeong Kim, Sangmin Bak, Euncheol Seo, Se Hyun Seong

**Affiliations:** a Gyeongsangbuk-do Agricultural Research and Extension Services, Gumi Floriculture Research Institute, Gumi, Republic of Korea; b Gyeongsangbuk-do Agricultural Research and Extension Services, Cheongdo Peach Research Institute, Cheongdo, Republic of Korea; Queens College

## Abstract

The complete genome sequence of *Apricot pseudo-chlorotic leaf spot virus* (APCLSV) isolate YC2, a South Korean isolate, was determined. The complete genome sequence was 7,491 nucleotides long and has a poly(A) tail. The YC2 isolate has 95% identity with another South Korean isolate and about 83% identity with an Italian isolate.

## Abstract (precis)

The complete genome sequence of *Apricot pseudo-chlorotic leaf spot virus* (APCLSV) isolate YC2, a South Korean isolate, was determined. The complete genome sequence was 7,491 nucleotides long and has a poly(A) tail.

## ANNOUNCEMENT

*Apricot pseudo-chlorotic leaf spot virus* (APCLSV) is a member of the genus Trichovirus in the family *Betaflexiviridae*. Members of the genus Trichovirus have flexuous and elongated particles about 700 nm long. APCLSV has a single-stranded positive-sense RNA genome with three open reading frames (ORFs). APCLSV was first reported in Japanese plums (Prunus salicina) in Italy and then has been reported in Spain, France, Hungary, Turkey, Jordan, Australia, China, the Czech Republic, and South Korea (1–6). Sus2, the first reported isolate of APCLSV, was isolated from Japanese plums showing severe stem pitting and stem grooving symptoms. Also, in a number of cases, APCLSV has been identified in Prunus spp., causing stem pitting or stem grooving symptoms on the wood, but we did not observe significant symptoms in the collected peach samples. Finally, there is no evidence of economic damage in South Korea caused by these viruses.

In this study, peach leaf samples were collected in peach orchards in Gyeongsangbuk-do Province, South Korea. Total RNA was extracted from leaves using the easy-spin total RNA extraction kit (iNtRON Biotechnology, Inc., South Korea), and then cDNA was synthesized using the SuperiorScript III cDNA synthesis kit (Enzynomics Co., Ltd., South Korea). An oligo(dT)_18_ primer was used as the initiator of synthesis. This cDNA was used as the template to determine the complete genome sequence of APCLSV. PCRs were performed using six designed primer pairs (Table 1) based on two complete genome sequences reported in Italy and South Korea (1, 6). As a result, six overlapping PCR products were amplified using Lamp *Taq* DNA polymerase (BioFact Co., Ltd., South Korea). To determine the complete genome sequence, the 5′ and 3′ untranslated regions (UTRs) of the genome were amplified using the SMARTer rapid amplification of cDNA ends (RACE) 5′/3′ kit (Clontech Laboratories, Inc., USA). The amplified fragments were cloned using the All In One PCR cloning kit (BioFact) and sequenced (Macrogen, Inc., South Korea). All determined nucleotide sequences were assembled using the DNAMAN version 7.0.8.2 software.

**TABLE 1:** Primers used in PCR for full-length sequencing of *Apricot pseudo-chlorotic leaf spot virus*

Primer name	Primer sequence (5′ to 3′)	Locus (nucleotides)^*a*^	Expected size (bp)
F210	GAGGAAGAATTGAAGGTGAAC	210–230	1,795
R2004	GATTTGCAAGTCCTTCGACC	2004–1985	
F1950	GGTTTCAAATCAAATGGTGAGG	1950–1971	1,435
R3384	CTACCAAAATGCCCCTTGAC	3384–3365	
F2429	GGAGTGTTATGATGATGATGG	2429–2449	1,735
R4163	GAGTGGGACTGATGACATG	4163–4145	
F3865	CAGCATGATAGAGAAGGAGC	3865–3884	2,007
R5871	CTGAGTCCTTGATGTCCTC	5871–5853	
F5414	GCTGGAGATGATATGTGTGC	5414–5433	1,182
R6595	GCTGTAGACCTAACGTCAG	6595–6577	
F5901	CCAATGACTGAAATCAACCAG	5901–5921	1,521
R7421	CTCCTTTGATAAACTGGGAC	7421–7396	

a Locus based on complete genome of APCLSV BC isolate (GenBank accession number KY310579).

The complete genome sequence of the APCLSV isolate YC2 contains 7,491 nucleotides (nt), and it is predicted to have three open reading frames (ORFs), similar to other trichoviruses, using the NCBI ORF Finder. The 5′ and 3′ UTRs were composed of 107 and 152 nt, respectively. A BLASTN search showed the closest identity of about 95% with the APCLSV BC isolate (6); the YC2 and BC isolates were reported in South Korea. However, the BC isolate was sequenced using multiple samples simultaneously by next-generation sequencing (NGS), so the effects of APCLSV, such as symptoms and economic damage caused by it, could not be specified. Also, the YC2 isolate has no evidence of environmental impact. In the previous study, no symptoms were observed on the peach tree in which APCLSV was detected, but stem pitting, stem grooving, and graft incompatibility problems were found on plum and apricot trees (7). To assess the impact of APCLSV, it is necessary to investigate other hosts of APCLSV.

### Data availability.

The complete genome sequence of APCLSV isolate YC2 was deposited in GenBank under the accession number MG879477.
